# Semiaquatic mammals might be intermediate hosts to spread avian influenza viruses from avian to human

**DOI:** 10.1038/s41598-019-48255-5

**Published:** 2019-08-12

**Authors:** Ping Zhao, Lingsha Sun, Jiasheng Xiong, Chuan Wang, Liang Chen, Pengfei Yang, Hao Yu, Qingli Yan, Yan Cheng, Lufang Jiang, Yue Chen, Genming Zhao, Qingwu Jiang, Chenglong Xiong

**Affiliations:** 1 Department of Epidemiology, School of Public Health, Fudan University, Shanghai, China; 2 School of Public Health, Fudan University, Key Laboratory of Public Health Safety, Ministry of Education, Shanghai, China; 3 College of Marine Science, Shandong University, Weihai, China; 4 The First Affiliated Hospital of Dalian Medical University, Dalian, China; 5 State Key Laboratory of Cardiovascular Disease, Fuwai Hospital, National Center for Cardiovascular Diseases, Chinese Academy of Medical Sciences and Peking Union Medical College, Beijing, China; 6 Huai’an Center for Disease Control and Prevention, Huai’an, China; 7 Hongze Center for Disease Control and Prevention, Hongze, China; 8 School of Epidemiology and Public Health, Faculty of Medicine, University of Ottawa, Ottawa, Canada

**Keywords:** Influenza virus, Ecological epidemiology

## Abstract

Avian influenza A viruses (AIVs) can occasionally transmit to mammals and lead to the development of human pandemic. A species of mammal is considered as a mixing vessel in the process of host adaptation. So far, pigs are considered as a plausible intermediate host for the generation of human pandemic strains, and are labelled ‘mixing vessels’. In this study, through the analysis of two professional databases, the Influenza Virus Resource of NCBI and the Global Initiative on Sharing Avian Influenza Data (GISAID), we found that the species of mink (*Neovison vison*) can be infected by more subtypes of influenza A viruses with considerably higher α-diversity related indices. It suggested that the semiaquatic mammals (riverside mammals), rather than pigs, might be the intermediate host to spread AIVs and serve as a potential mixing vessel for the interspecies transmission among birds, mammals and human. In epidemic areas, minks, possibly some other semiaquatic mammals as well, could be an important sentinel species for influenza surveillance and early warning.

## Introduction

Influenza A viruses (IAVs) belong to the family Orthomyxoviridae. Based on the antigenic properties of two surface glycoproteins hemagglutinin (HA) and neuraminidase (NA), IAVs are clustered into 18 HA (H1–H18) and 11 NA (N1–N11) subtypes^1–3^. The ecology of IAVs is complicated involving multiple host species and viral genes. So far, except that H17, H18, N10 and N11 were restrictively identified from bat samples in forms of H17N10 and H18N11^4^, all other subtypes viruses can circulate in avian species^1,2,5^, aquatic birds (or waterfowls) in particular, which are therefore considered the natural reservoir of IAVs^6–11^. Occasionally, avian influenza A viruses (AIVs) can transmit to mammals from avian species, which may lead to the development of human pandemic strains by direct or indirect transmission.

A successful transmission between species depends on both host and virus factors, and some period of adaptation of the virus to the new species. Many host factors interacting with the component proteins of IAVs have been identified and their role in the host range expansion and interspecies transmission has been clearly stated^8,9^. Several viral proteins of IAVs are also known to be responsible for host adaptation or interspecies transmission^6,12–18^, of which HA membrane protein is the major determinant for crossing the species barrier^8,19,20^. Binding to sialic acid receptor, HA initiates fusion of the viral envelope with the host cell membrane^5,21,22^. The sialic acid receptor can be linked to galactose by α2,6-linkages (SAα2,6 Gal) or α2,3-linkages (SAα2,3 Gal). It is generally believed that HAs of AIVs preferentially bind to SAα2,3 Gal on intestinal epithelial cells of aquatic birds, whereas the HAs of human IAVs prefer SAα2,6 Gal on tracheal epithelium. The adaptation of AIVs to human or other mammalian hosts (mammalian influenza A viruses are abbreviated as MIVs in this study) is connected with a switch in HA ability to bind SAα2,6 Gal instead of SAα2,3 Gal^20,23–28^. In pig tracheal epithelium, there exist both SAα2,3 Gal and SAα2,6 Gal; HA of both AIVs and human influenza viruses may find the receptors. Given these features, pigs are considered as a plausible intermediate host for the generation of human pandemic strains by gene reassortment^5,29–35^. This potential to generate novel influenza viruses has resulted in swine being labelled ‘mixing vessels’^36–38^.

The variety of AIVs combined with the high ability of adaptation constitutes the main risk factor for crossing the species barriers, but it is difficult to predict which virus might induce a human pandemic^8,39^. In order to identify precursor viruses of potential pandemics, an active surveillance and collecting of AIVs from different species, especially the species that can be served as mixing vessels, are crucial. In this study, through screening the host originations of all subtypes of HA and NA sequences available in public databases, we analyzed their host tropisms and attempted to provide the target hosts other than pigs for surveillance of influenza pandemics.

## Methods

### Screen and count the host originations of IAVs

As mentioned above, HA can initiate fusion of the viral envelope with the host cell membrane, which is the prerequisite for viral replication and transmission. While the balance between HA receptor-binding affinity and NA receptor destroying activity is critical for the efficient growth of IAVs, NA also contributes to influenza virus species specificity^40,41^. Therefore, HA and NA nucleotide sequences were analyzed in this study.

The host originations of HA and NA nucleotide sequences were screened and counted in two databases, the Influenza Virus Resource of NCBI (http://www.ncbi.nlm.nih.gov/genomes/FLU/aboutdatabase.html) and the Global Initiative on Sharing Avian Influenza Data (GISAID, http://platform.gisaid.org/epi3/frontend) by the end of March 12, 2018. NCBI was used as the main database while the latter was used as a supplementary under the set of *only GISAID uploaded isolates*. Two screen strategies were engaged in this study. Considering that large amount of isolates of IAV were not sequenced and submitted completely to the databases, the host originations of HA and NA sequences were screened separately. The other strategy that the host originations were screened and counted by subtypes of HxNy (x = 1, 2, 3…18, y = 1, 2, 3…11), was carried out when the preferential HA-NA balances of IAVs were taken into account.

### Measure for α-diversity indices of IAVs established from mammalian hosts

Microenvironment in host animal provides the material basis for the growth and proliferation of viruses; at the same time, antibodies and receptor types can also restrict the IAV infections. Relationship or interaction between the microenvironment of a host and viruses is somewhat similar to that between ecological environment and the populations living in it. When a host can be infected with different subtypes of IAVs, it is more likely to be a mixing vessel or natural reservoir for the viruses. Each subtype of HA or NA was regarded as a species population, while the sequence frequencies recorded in the databases were regarded as the observed individuals of the corresponding populations. We can use use some ecological indices such as species diversity, richness, and evenness of IAVs within a species of mammalian host to measure the complexity of a relationship between host microenvironment and IAVs^42^:

1. Margalef (1951, 1957, and 1958) index (focuses on richness)
  $${{D}}_{r}=({\rm{S}}-1)/\mathrm{lnN}$$
  S is the total subtype number of HA or NA that established from a species of mammalian hosts, and N is the total sequence frequency of HA or NA established from this host species.
2. Simpson’s index (focuses on dominance)
  $${{D}}_{s}=1-{{\rm{\Sigma }}\mathrm{Pi}}^{2}$$
  Pi refers to the ratios of the number of ni subtype of HA or NA to the total sequence frequency of HA or NA established from a species of mammalian hosts, i.e., Pi = ni/N.
3. Shannon-wiener diversity index
  $$H^{\prime} =-\,{\rm{\Sigma }}\mathrm{PilnPi}$$
  The meaning of Pi is the same as above.
4. Pielou evenness index

$$E={\rm{H}}/{\rm{lnS}}$$

H is the observed species diversity index, which equals to Shannon-wiener index *H*′, i. e., *H* = *H*′ = −ΣPilnPi.

### Sequence analyses for focused HAs and NAs of AIVs

A species of mammalian host may have the tendency to become a mixing vessel or natural reservoir for human IAVs or other MIVs if they can be infected with a large number of subtypes of IAVs, AIVs in particular. We further studies the species of mammalian hosts infected with the IAVs that had the highest richness, diversities, and evenness. After consulting the information in GenBank and GISAID in detail, tracking references, and removing repeated sequence submissions, each of the HA and NA sequences of AIVs was analyzed by using the Basic Local Alignment Search Tool (BLAST, https://blast.ncbi.nlm.nih.gov/Blast.cgi) with the set of *Max target sequences* being 1000, and then, every 1000 sequences were downloaded to a local computer. After alignment by FFT-NS-2 methods in multiple alignment program for amino acid or nucleotide sequences (MAFFT version 7, https://mafft.cbrc.jp), they were translated and compared by the MegAlign module of the Lasergene 7.0 software. For HAs, the parts of sequences encoding the signal peptides were cut off beforehand corresponding to each reference sequence of the respective subtype (https://www.ncbi.nlm.nih.gov/refseq). The comparisons were carried out between each sequence of HA or NA and its 999 most similar sequences, and variations on the sites that are known as being relevant to the host tropism of IAVs were focused on^3,23,24,39,43–51^.

### Ethics approval

This study is a serial of phylogenetic analyses based on large scale of existing gene sequences; all these sequences can be searched and downloaded from two public databases, the NCBI Influenza Virus Sequence Database and the Global Initiative on Sharing Avian Influenza Data (GISAID) database. No institutional review board approval was required from the research ethics committee of School of Public Health, Fudan University, and animals’ ethics approval was applicable neither.

## Results

From H1 to H18, and from N1 to N11, the ratios of sequences with mammalian host origination to those with avian host origination are displayed in Supplementary Figs 1, 2 and Supplementary Tables 1–3. Except for 65 sequences (33 HA and 32 NA) that were labeled as mammalian origination but no definite species records, 26 species of nonhuman mammal hosts of IAVs were retrieved from the databases. Further checking confirmed that the hosts labeled as feline are cats rather than the taxonomic family of feline. Bovine and mouse had entries but no sequence records. Thus, 23 species of nonhuman mammals were included for the subsequent analysis.

The mammalian species of bat, boar, camel, canine, cat, equine, ferret, mink, muskrat, seal, swine, and whale can be infected by more than one subtype of IAVs. For a long time, swine is considered as a mixing vessel for reassortment or recombination of IAVs. Although isolates established from swine are indeed abundant, the subtypes of them are restricted mainly to MIVs, of which, H1, H3, N1 and N2 account for the overwhelming majority (99·18% of HAs and 99·58% of NAs). The α-diversity related indices including the Shannon-wiener index, the Simpson’s diversity index, the Margalef richness, and the Pielou evenness and they were 0.88, 0.38, 0.94 and 0.27 for HAs that derived from swine, and were 1·00, 0·49, 0·64, and 0·36 for NAs. For HAs, the indices were even lower in swine as compared with those in cat, ferret, camel, bat and muskrat, and for NAs, they were not higher in swine than those in cat and camel. It seemed that swine can only be infected with limited subtypes of IAVs, and sporadic infections caused by subtypes other than H1N1, H1N2 and H3N2 occasionally occurred by chance of accidental spillover. The same happened in dogs and horses. Although the sequences of HA and NA established from them were abundant enough, the subtypes of IAVs were restricted to one or more specific subtypes of MIVs, of which H3N8 and H3N2 accounted for the overwhelming majority.

**Figure 1:**
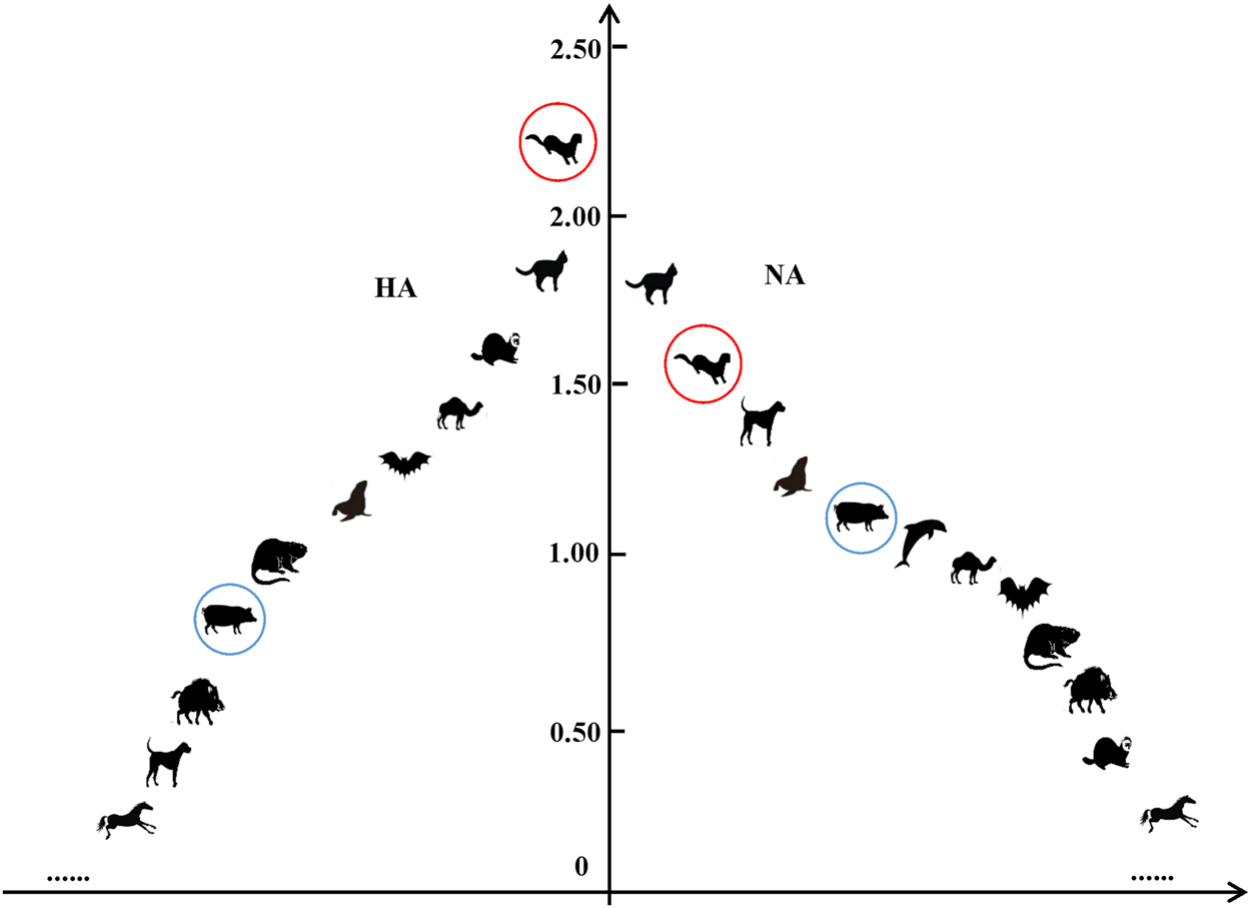
Shannon-wiener index of IAVs’ HA and NA derived from different mammals^*^. *Only those are greater than zero are shown. As for HA (left side), from high to low are mink, cat, ferret, camel, bat, seal, muskrat, swine, boar, canine, equine, in turn; as for NA (right side), they are cat, mink, canine, seal, swine, whale, camel, bat, muskrat, boar, ferret, equine, respectively.

Interestingly, a neglected mammalian host, mink, was infected by more subtypes of IAVs. Isolates including both MIVs (H3N2 and H1N1) and AIVs (H5N1, H9N2, and H10N4), had considerably higher α-diversity related indices. The Shannon-wiener index, the Simpson’s diversity index, the Margalef richness, and the Pielou evenness were 2·20, 0·77, 1·56, and 0·95 for HAs, and 1·46, 0·61, 0·81, and 0·92 for NAs. The α-diversity related indices of HAs and NAs derived from different mammalian hosts are displayed in Table 1 and Fig. 1.

**Table 1:** The α diversity related indices of HA and NA derived from mammalian hosts.

	HA						NA					
	*Ds*	*Dr*	*H*′	*E*	S	N	*Ds*	*Dr*	*H*	*E*	S	N
mink	0·77	1·56	2·20	0·95	5	13	0·61	0·81	1·46	0·92	3	12
cat	0·68	0·85	1·79	0·90	4	34	0·63	0·56	1·51	0·95	3	36
ferret	0·62	1·14	1·59	0·80	4	14	0·26	0·39	0·62	0·62	2	13
camel	0·50	1·44	1·00	1·00	2	2	0·50	1·44	1·00	1·00	2	2
bat	0·48	0·62	0·97	0·97	2	5	0·48	0·62	0·97	0·97	2	5
muskrat	0·44	0·91	0·92	0·92	2	3	0·44	0·91	0·92	0·92	2	3
swine	0·38	0·94	0·88	0·27	10	13841	0·49	0·64	1·00	0·36	7	11747
seal	0·30	0·92	0·97	0·42	5	78	0·57	1·36	1·38	0·72	5	19
boar	0·28	0·56	0·65	0·65	2	6	0·44	0·56	0·92	0·92	2	6
canine	0·09	0·64	0·32	0·14	5	520	0·59	0·38	1·40	0·88	3	188
equine	0·01	0·36	0·06	0·03	4	4075	0·10	0·51	0·32	0·16	4	361
whale	0	0	0	/	1	2	0·50	1·44	1·00	1·00	2	2
tiger	0	0	0	/	1	17	0	0	0	/	1	17
civet	0	0	0	/	1	4	0	0	0	/	1	2
raccoon dog	0	0	0	/	1	2	0	0	0	/	1	2
cheetah	0	0	0	/	1	2	0	0	0	/	1	2
anteater	0	/	0	/	1	1	0	/	0	/	1	1
leopard	0	0	0	/	1	6	0	/	0	/	1	1
lion	0	/	0	/	1	1	0	/	0	/	1	1
marten	0	/	0	/	1	1	0	/	0	/	1	1
panda	0	/	0	/	1	1	0	/	0	/	1	1
pika	0	0	0	/	1	6	0	0	0	/	1	6
bear	0	/	0	/	1	1	0	/	0	/	1	1

Fourteen HA and thirteen NA sequences were found to be established from minks, of which nine pairs of HA and NA were of the typical AIVs, including two H10N4, three H5N1, and four H9N2. BLAST analysis showed that one variation of G212R in two strains of H10N4, and one variation of N173H in the isolate China/01/2014 (H9N2) might involve the binding epitopes of the globular head of HA protein, and the rest variations did not site in the known binding epitopes of the host cell receptor (Table 2).

**Table 2:** Variations of HAs and NAs of AIVs established from minks compared to majorities.

Strain	HA		NA	
	Access No.	Variation	Access No.	Variation
Sweden/3900/1984(H10N4)	GQ176144	E23V, A92T, E97G, I112T, T117A, N137D, L143V, S150D, *G212R*, L245R, V525A	GQ176142	E75D, R79S, N/T271P, V/E328M
Sweden/E12665/1984(H10N4)	M21646	the same as above	AY207530	the same as above
Sweden/V907/2006(H5N1)	EU889075	E502G	EU889101	/
Shandong/F6/2013(H9N2)	KM576103	K276E, G294E, D366G, Y414C, S486P, E501G	KM576104	P55S, N61S, N218H, K293N
Shandong/F10/2013(H9N2)	KM576111	the same as above	KM576112	the same as above
China/01/2014(H9N2)	MF996800	*N173H*	MF996801	K77N, K196T, G245E, G267R, N/D325S
China/02/2014(H9N2)	MF996789	V(−14)I, G230D, R537G	MF996791	Q90K, K/N140R, I202V, Y281H, N355D, S397N
China/G/2015(H5N1)	KX867865	I(−12)T, K162E, P194L, I375M, E502G	KX867867	Q45H, F302L
China/XB/2015(H5N1)	KX867873	I374M	KX867875	/

## Discussion

Swine is an important host for IAVs for reasons of being involved in genetic reassortment and interspecies transmission. In swine population, H1N1, H3N2, H1N2 viruses are circulating worldwide, and most swine influenza viruses (SIVs) are reassortants originated from human, avian and swine influenza viruses^24,52,53^. However, our study indicated that the spillover infections of swine occurred only occasionally. Rare spillover infections were similar for dogs, horses and cats.

Remarkably, our results suggest that mink should be taken more seriously in influenza surveillance. Mink (*Neovison vison*) is a semiaquatic mammal (or riverside mammal, mammals occurring close to the water and sometimes within it, such as *Neovison vison*, *Lutra lutra*, Delphinidae, and Phocidae) species of the genus *Mustela* of the family Mustelidae; there are 15 subspecies of mink widely distributed in the Americas or being introduced into other continents^54^. IAVs including both AIVs (H9N2, H5N1, and H10N4) and MIVs (H3N2 and H1N1), were isolated from minks with the highest species/subtype diversities, richness and evenness. Influenza A has caused several outbreaks in minks^55–58^. The same strain of MIV or AIV can be repeatedly established during one outbreak^55,58^, and different subtypes of AIVs also can be isolated from an outbreak in the same period and same breeding farm^59,60^. All these testimonies prove the susceptibility of mink to IAVs and the transmission features within the populations. Peng *et al*. and Yu *et al*. have reported that receptors in tracheal epithelium of mink are mainly linked to SAα2,6 Gal, but receptors of SAα2,3 Gal and SAα2,6 Gal are detected equally or with predominance to SAα2,3 Gal in gastrointestinal mucosa of it^58,61^. As we know, HAs of AIVs preferential receptors of SAα2,3 Gal are coincidentally on intestinal epithelial cells of aquatic birds^26,31,62^. Such a molecular basis of the existence of both AIVs and MIVs specific receptors within minks, as well as their characteristic distribution, imply that minks not only could infect with MIVs by intra-tracheal inoculation or horizontal transmission from other minks within populations, but also could infect with AIVs either by eating (preying or feeding) on virus-infected birds or by faecal-oral route within habitat environment^63,64^. These findings suggest minks may be another intermediate host to spread the virus from wild waterfowls to human. Mink infection may contribute to the adaptation of AIVs to human and other mammals by genetic reassortment or other mechanisms.

**Figure 2:**
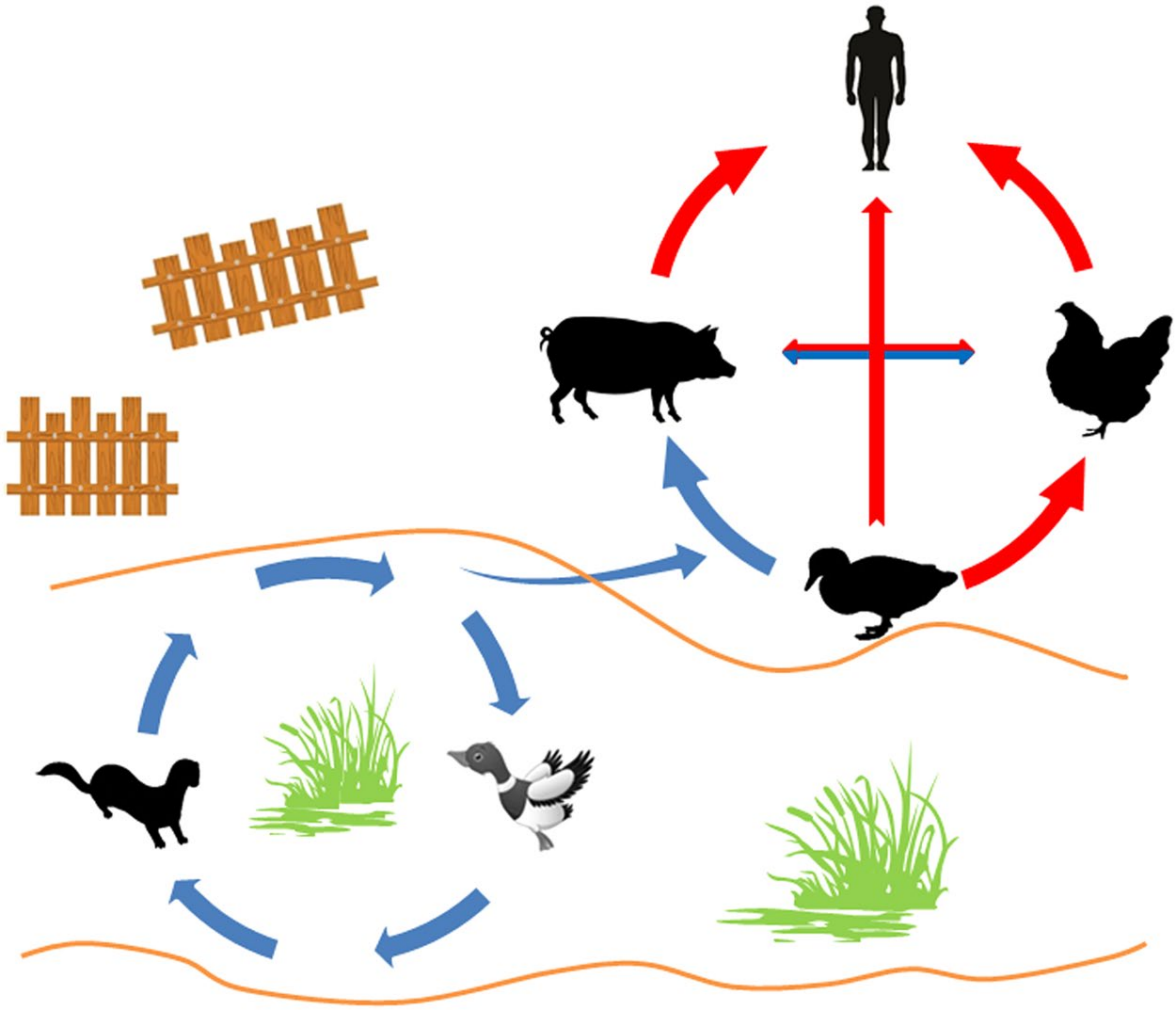
The illustration of adaptation and transmission of Human AIVs. An adaptation from AIVs to human AIVs includes two circulations, the aquatic habitat circulation and the land habitat circulation. In the aquatic habitat circulation, AIVs are transmitting, mutating, and adapting between aquatic birds and minks (as well as other semiaquatic mammals). This adaptation may or may not change their infectivity to avian, but can significantly increase the infectivity to human and terrestrial mammals. Poultries such as duck, goose can be infected through contacting with epidemic water. In a free stall barn system, usually in some areas of South Asia, Southeast Asia, Southern and Eastern China, it will inevitably lead to a land habitat circulation including human beings. The blue pathway is transmitted by faecal-oral route, while the red one is transmitted by intra-tracheal inoculation. The conception for this scene is partly based on the observation of daily lives; e.g., in rural areas of South Asia, Southeast Asia, Southern and Eastern China, pigs and poultries, in particular chick, are often observed to eat each other’s feces. Pigs also eat duck feces, but ducks seldom eat pig feces; and partly based on the available reports, e.g., human infected by a human-adapted AIV from the live poultry market was often reported in China.

From the viewpoint of niche, habitats of riverside mammals such as minks and wild waterfowls overlap each other, which greatly facilitate the interspecies transmission among them. Possibly, some other species of riverside mammals, in addition to terrestrial and domesticated pigs, might also have this potential. Waterfowls have long been considered natural gene pools for IAVs. While the receptors on the surface of gastrointestinal mucosa can recur the infections caused by AIVs within waterfowls, minks may be of significance in sustaining IAVs’ genes and the species may be both a mixing vessel and natural reservoir for IAVs. Co-infection greatly increases the chances of generating novel viruses through genetic reassortment or recombination, which can introduce a novel subtype of IAV in human population. In a free stall barn system, usually in some areas of South Asia, Southeast Asia, Southern and Eastern China, the traditional methods of free-range or outdoor breeding always exacerbates the risk of infection of poultry and backyard livestock through contact with contaminated water or feces^65–67^. The emergence of novel IAVs can lead to a rapid epidemic within terrestrial animals or human. Circulation of IAVs among minks or other riverside mammals, waterfowl, domestic poultry, terrestrial mammals and human is illustrated in Fig. 2.

This study demonstrates that mink (*Neovison vison*) might be a potential mixing vessel or intermediate host for the generation of novel human IAVs. Minks, possibly some other semiaquatic mammals (riverside mammals) as well, might play a pivotal role in the process of adapting and transmitting AIVs to human and other terrestrial animals. The significances of mink and other riverside mammal hosts in influenza surveillance and early warning should be paid an attention. In epidemic areas, mink should be considered as one of important sentinel species of hosts for influenza surveillance.

There are several limitations of our study should be mentioned. In this study, we only used the existing databases with no additional laboratory evidence. Secondly, the number of IAVs established from the mammalian species here including those in minks is still small. Hence, our conclusions need to be consolidated.

## Supplementary information

Supplementary figures and tables
