# Supplementary material for: Semiaquatic mammals might be intermediate hosts to spread avian influenza viruses from avian to human
Source: Sci Rep. 2019 Aug 12;9:11641. doi: 10.1038/s41598-019-48255-5 (PMC6690891; doi:10.1038/s41598-019-48255-5)
Supplement: Supplementary file 1 — Supplementary figures and tables [file 41598_2019_48255_MOESM1_ESM.pdf]

Semiaquatic mammals might be intermediate hosts to spread avian influenza viruses from avian to human

Ping Zhao, Lingsha Sun, Jiasheng Xiong, Chuan Wang, Liang Chen, Pengfei Yang, Hao Yu, Qingli Yan, Yan Cheng, Lufang Jiang, Yue Chen, Genming Zhao, Qingwu Jiang & Chenglong Xiong

Supplementary Fig. 1 The sequences of HAs and NAs with human, swine, other nonhuman mammals, and avian host originations

a, and b: constituent ratios of HA and NA sequences derived from human, swine, other nonhuman mammals, and avian host;

c, and d: numbers of HA and NA sequences with human, swine, other nonhuman mammals, and avian host originations.

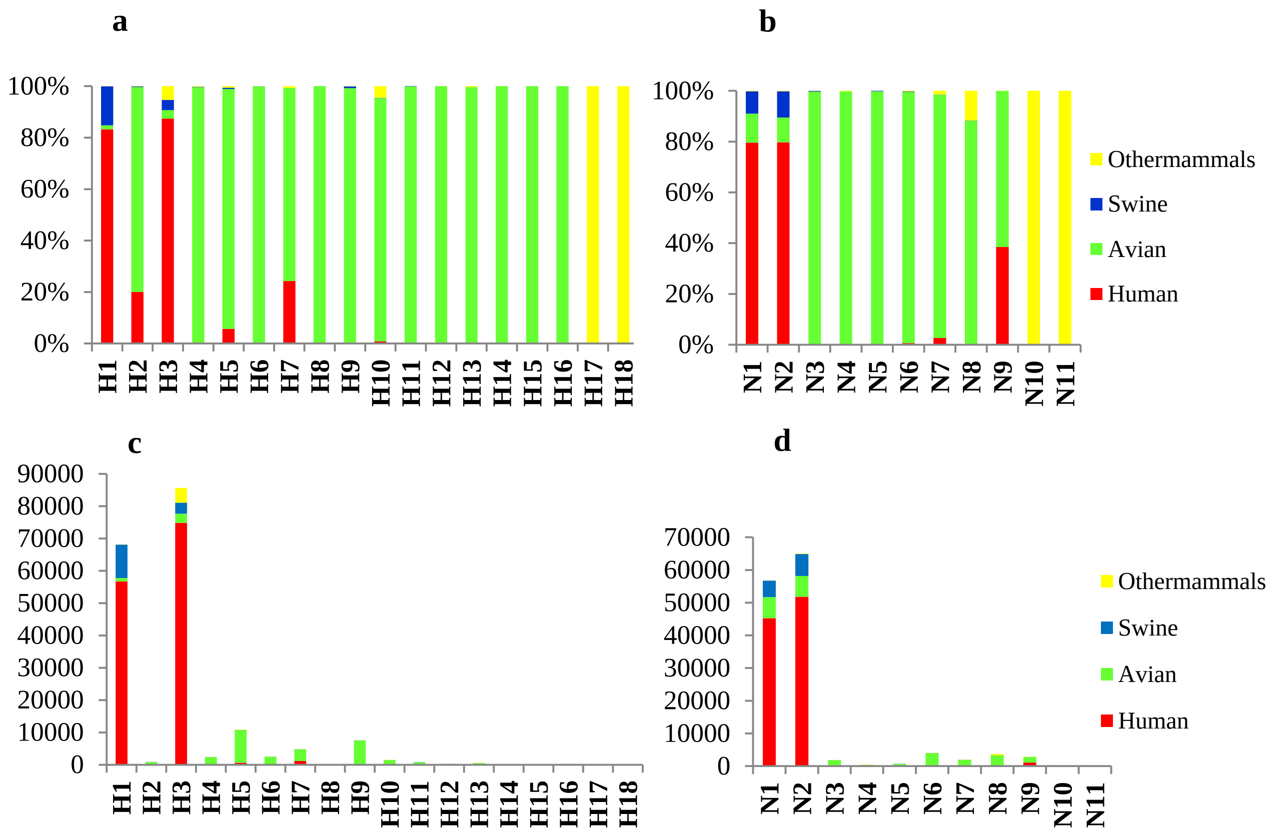

a
b
c
d
100%
80%
60%
40%
20%
0%
H1 H2 H3 H4 H5 H6 H7 H8 H9 H10 H11 H12 H13 H14 H15 H16 H17 H18
N1 N2 N3 N4 N5 N6 N7 N8 N9 N10 N11
90000
80000
70000
60000
50000
40000
30000
20000
10000
0
Othermammals
Swine
Avian
Human

Supplementary Fig. 2 The balances of HAs and NAs in the top five common subtype s of MIVs and AIVs*

a, HAs of the top five common MIVs and AIVs and the distribution of their corresponding NA subtypes;

b, NAs of the top five common MIVs and AIVs and the distribution of their corresponding HA subtypes.

*Except H2, common subtype refers to those subtypes that have more than 1000 sequence records in the database; H2 had ever caused pandemics in Asia, and its record is currently 813 in the two databases.

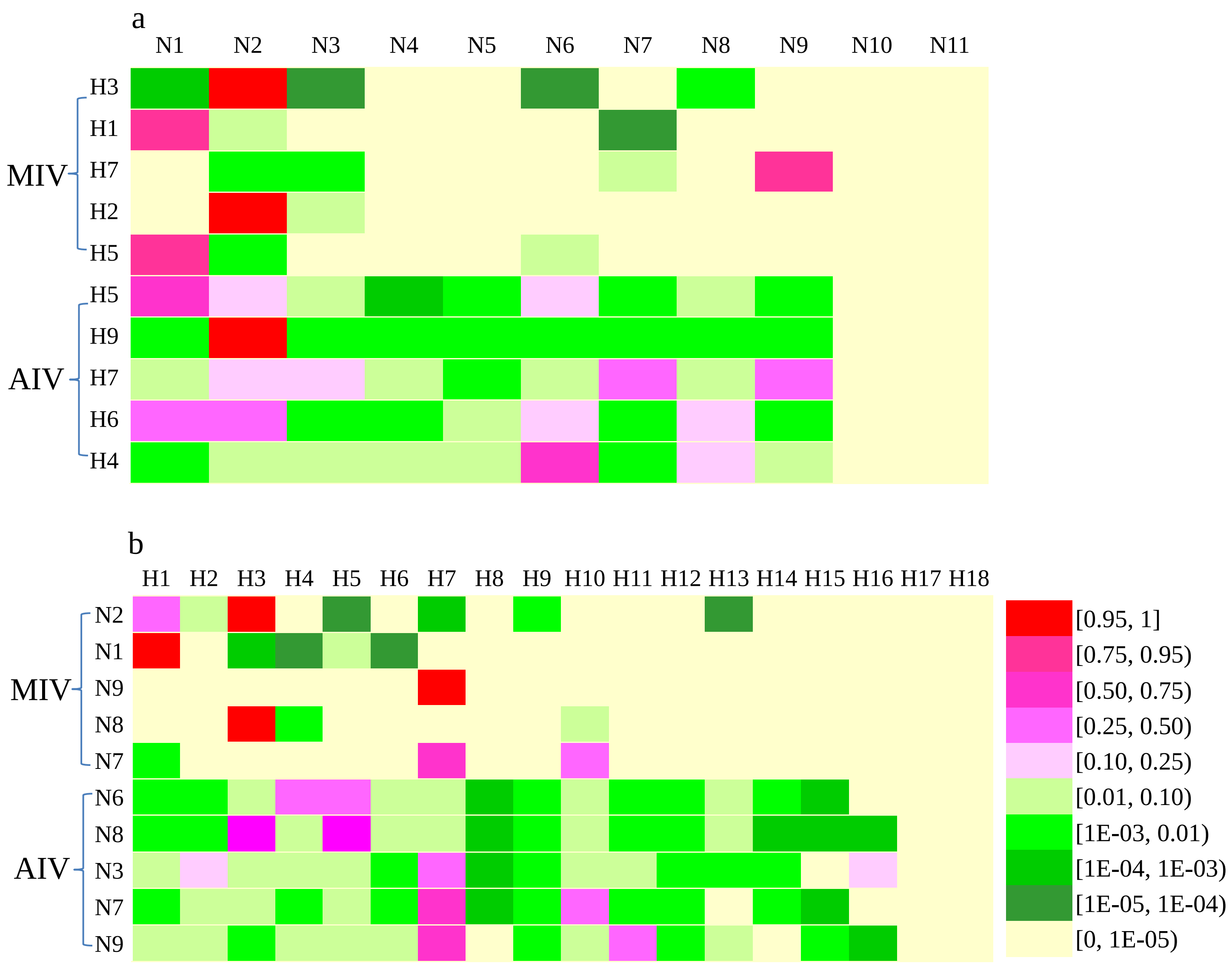
a
N1 N2 N3 N4 N5 N6 N7 N8 N9 N10 N11
MIV
H3 H1 H7 H2 H5
AIV
H5 H9 H7 H6 H4
b
H1 H2 H3 H4 H5 H6 H7 H8 H9 H10 H11 H12 H13 H14 H15 H16 H17 H18
MIV
N2 N1 N9 N8 N7
AIV
N6 N8 N3 N7 N9
[0.95, 1]
[0.75, 0.95)
[0.50, 0.75)
[0.25, 0.50)
[0.10, 0.25)
[0.01, 0.10)
[1E-03, 0.01)
[1E-04, 1E-03)
[1E-05, 1E-04)
[0, 1E-05)

Supplementary Tab. 1 The sequences of HAs and NAs with human, swine, other nonhuman mammals, and avian host originations

		Human		Avian		Swine		Other mammals	
		**amount**	**ratio**	**amount**	**ratio**	**amount**	**ratio**	**amount**	**ratio**
**HA**	**H1**	56720	0.83216	1074	0.01576	10332	0.15158	34	0.00050
	**H2**	160	0.20075	634	0.79548	2	0.00251	1	0.00125
	**H3**	74879	0.87407	2827	0.03300	3396	0.03964	4565	0.05329
	**H4**	0	0.00000	2342	0.99575	5	0.00213	5	0.00213
	**H5**	614	0.05653	10132	0.93279	49	0.00451	67	0.00617
	**H6**	1	0.00040	2466	0.99838	2	0.00081	1	0.00040
	**H7**	1159	0.24247	3586	0.75021	1	0.00021	34	0.00711
	**H8**	0	0.00000	213	0.00000	0	1.00000	0	0.00000
	**H9**	28	0.00372	7443	0.98845	52	0.00691	7	0.00093
	**H10**	12	0.00803	1414	0.94582	1	0.00067	8	0.04548
	**H11**	0	0.00000	779	0.99872	1	0.00128	0	0.00000
	**H12**	0	0.00000	263	1.00000	0	0.00000	0	0.00000
	**H13**	0	0.00000	534	0.99627	0	0.00000	2	0.00373
	**H14**	0	0.00000	38	1.00000	0	0.00000	0	0.00000
	**H15**	0	0.00000	25	1.00000	0	0.00000	0	0.00000
	**H16**	0	0.00000	275	1.00000	0	0.00000	0	0.00000
	**H17**	0	0.00000	0	0.00000	0	0.00000	3	1.00000
	**H18**	0	0.00000	0	0.00000	0	0.00000	2	1.00000
**NA**	**N1**	45232	0.79604	6494	0.11429	5006	0.08810	89	0.00157
	**N2**	51821	0.79726	6351	0.09771	6723	0.10343	104	0.00160
	**N3**	2	0.00110	1805	0.99614	4	0.00221	1	0.00055
	**N4**	0	0.00000	408	0.99756	0	0.00000	1	0.00244
	**N5**	0	0.00000	625	0.99840	1	0.00160	0	0.00000
	**N6**	24	0.00604	3928	0.98892	9	0.00227	11	0.00277
	**N7**	50	0.02665	1797	0.95789	1	0.00053	28	0.01493
	**N8**	10	0.00269	3266	0.87938	3	0.00081	435	0.11712
	**N9**	1071	0.38511	1709	0.61453	0	0.00000	1	0.00036
	**N10**	0	0.00000	0	0.00000	0	0.00000	5	1.00000
	**N11**	0	0.00000	0	0.00000	0	0.00000	2	1.00000

Supplementary Tab. 2a The balances of HAs and NAs in the top five common subtype s of MIVs -according to HA

	**H3**		**H1**		**H7**		**H2**		**H5**	
	**amount**	**ratio**	**amount**	**ratio**	**amount**	**ratio**	**amount**	**ratio**	**amount**	**ratio**
N1	48	0.00087	49505	0.93318	0	0	0	0	603	0.94366
N2	54588	0.99110	3544	0.06680	9	0.00783	145	0.98639	4	0.00626
N3	3	0.00005	0	0	2	0.00174	2	0.01361	0	0
N4	0	0	0	0	0	0	0	0	0	0
N5	0	0	0	0	0	0	0	0	0	0
N6	1	0.00002	0	0	0	0	0	0	32	0.05008
N7	0	0	1	0.00002	68	0.05918	0	0	0	0
N8	438	0.00795	0	0	0	0	0	0	0	0
N9	0	0	0	0	1070	0.93124	0	0	0	0
N10	0	0	0	0	0	0	0	0	0	0
N11	0	0	0	0	0	0	0	0	0	0

Supplementary Tab. 2b The balances of HAs and NAs in the top five common subtype s of AIVs -according to HA

	**H5**		**H9**		**H7**		**H6**		**H4**	
	**amount**	**ratio**	**amount**	**ratio**	**amount**	**ratio**	**amount**	**ratio**	**amount**	**ratio**
N1	4624	0.60794	23	0.00775	240	0.07600	527	0.25583	18	0.00894
N2	884	0.11622	2886	0.97204	340	0.10766	817	0.39660	175	0.08693
N3	159	0.02090	11	0.00370	670	0.21216	13	0.00631	24	0.01192
N4	6	0.00079	3	0.00101	44	0.01393	12	0.00583	23	0.01143
N5	64	0.00841	15	0.00505	6	0.00190	97	0.04709	25	0.01242
N6	1118	0.14699	6	0.00202	58	0.01837	342	0.16602	1450	0.72032
N7	10	0.00131	6	0.00202	966	0.30589	6	0.00291	11	0.00546
N8	715	0.09400	5	0.00168	38	0.01203	232	0.11262	261	0.12966
N9	26	0.00342	14	0.00472	796	0.25206	14	0.00680	26	0.01292
N10	0	0	0	0	0	0	0	0	0	0
N11	0	0	0	0	0	0	0	0	0	0

Supplementary Tab. 3a The balances of HAs and NAs in the top five common subtype s of MIVs -according to NA

	N2		N1		N9		N8		N7	
	**amount**	**ratio**	**amount**	**ratio**	**amount**	**ratio**	**amount**	**ratio**	**amount**	**ratio**
H1	3806	0.04806	61836	0.98803	0	0	0	0	1	0.00667
H2	159	0.00201	0	0	0	0	0	0	0	0
H3	75131	0.94865	52	0.00083	0	0	455	0.97639	0	0
H4	0	0	1	0.00002	0	0	1	0.00215	0	0
H5	4	0.00005	694	0.01109	0	0	0	0	0	0
H6	0	0	2	0.00003	0	0	0	0	0	0
H7	9	0.00011	0	0	1100	1.00000	0	0	82	0.54667
H8	0	0	0	0	0	0	0	0	0	0
H9	87	0.00110	0	0	0	0	0	0	0	0
H10	0	0	0	0	0	0	10	0.02146	67	0.44667
H11	0	0	0	0	0	0	0	0	0	0
H12	0	0	0	0	0	0	0	0	0	0
H13	2	0.00003	0	0	0	0	0	0	0	0
H14	0	0	0	0	0	0	0	0	0	0
H15	0	0	0	0	0	0	0	0	0	0
H16	0	0	0	0	0	0	0	0	0	0
H17	0	0	0	0	0	0	0	0	0	0
H18	0	0	0	0	0	0	0	0	0	0

Supplementary Tab. 3b The balances of HAs and NAs in the top five common subtype s of AIVs -according to NA

	N6		N8		N3		N7		N9	
	**amount**	**ratio**	**amount**	**ratio**	**amount**	**ratio**	**amount**	**ratio**	**amount**	**ratio**
H1	38	0.00999	24	0.00723	76	0.03936	8	0.00510	56	0.03590
H2	9	0.00237	19	0.00573	280	0.14500	26	0.01658	69	0.04423
H3	299	0.07858	1526	0.45992	33	0.01709	16	0.01020	14	0.00897
H4	1502	0.39474	271	0.08168	27	0.01398	11	0.00702	26	0.01667
H5	1170	0.30749	913	0.27517	192	0.09943	16	0.01020	43	0.02756
H6	353	0.09277	279	0.08409	14	0.00725	6	0.00383	16	0.01026
H7	60	0.01577	42	0.01266	815	0.42206	804	0.51276	811	0.51987
H8	1	0.00026	2	0.00060	1	0.00052	1	0.00064	0	0
H9	7	0.00184	6	0.00181	11	0.00570	7	0.00446	14	0.00897
H10	67	0.01761	94	0.02833	129	0.06680	656	0.41837	30	0.01923
H11	18	0.00473	12	0.00362	68	0.03521	9	0.00574	442	0.28333
H12	6	0.00158	6	0.00181	5	0.00259	5	0.00319	4	0.00256
H13	267	0.07017	118	0.03556	7	0.00363	0	0	23	0.01474
H14	6	0.00158	1	0.00030	13	0.00673	2	0.00128	0	0
H15	2	0.00053	3	0.00090	0	0	1	0.00064	11	0.00705
H16	0	0	2	0.00060	260	0.13465	0	0	1	0.00064
H17	0	0	0	0	0	0	0	0	0	0
H18	0	0	0	0	0	0	0	0	0	0
